# Place and space in relation to childbirth: a critical interpretive synthesis

**DOI:** 10.1080/17482631.2019.1667143

**Published:** 2020-10-25

**Authors:** Ing-Marie Carlsson, Ingrid Larsson, Henrika Jormfeldt

**Affiliations:** Department of Health and Welfare, Halmstad University, Halmstad, Sweden

**Keywords:** Childbirth, critical interpretive synthesis, place, space, qualitative studies

## Abstract

**Background**: In nursing and midwifery, the concept of environment is considered a meta-concept. Research findings suggest that the location is not the only important factor, as both place and space influence the practices of midwives. Moreover, research on the geography of health suggests a connection between place and health that could be extended to reproductive health. Therefore, to move beyond and expand traditional research expressions, it is beneficial to illuminate the concepts of place and space in relation to childbirth.

**Purpose**: This study was undertaken to produce a synthesis of previous qualitative research of issues in childbirth in relation to the concepts of place and space.

**Method**: In this Critical Interpretive Synthesis (CIS), four electronic databases; CINAHL, Medline, PsycINFO and Sociological abstracts, were used for the literature search. In total 734 papers were screened, and 27 papers met the final inclusion criteria after assessment.

**Results**: The synthesis reveals a need to create a space for childbirth underpinned by four aspects; a homely space, a spiritual space, a safe space, and a territorial space.

**Conclusion**: Findings from this review will provide a basis for useful dialogue in midwifery education and in clinical settings.

## Background

The provision of good and qualitative antenatal care is vital to childbirth. It is also a global goal for promoting the best maternal and children’s health and well-being (World Health Organization [WHO], 2011). However, antenatal care varies worldwide due to the differences in health policies and legalizations between countries. Therefore, there are differences in organization of care, and different “models of care”, i.e. which profession is the lead healthcare professional for providing care during childbirth (Sandall, Soltani, Gates, Shennan, & Devane, 2016; Symon et al., 2016). Rooks (1999) has highlighted two theoretical models of care during childbirth. The first model is the medical model, which is characterized by the idea that childbirth is a risk and that birth can only be defined as normal in retrospect. According to this model, the hospital is the safest place, since medical care and interventions can be performed if complications occur during childbirth. The second theoretical model is the midwifery model (Rooks, 1999). This model focuses on and supports the normalcy in childbirth and has a woman-centred approach (Kennedy, 2000; Rooks, 1999). The midwifery model acknowledges that although most births can proceed without unnecessary medical interventions, focusing on normalcy does not exclude medical treatment if needed (Olsen & Clausen, 2012). The midwifery model is in line with the strategy of the International Confederation of Midwives (International Confederation of Midwives [ICM], 2014) for supporting normal birth. ICM (2014) also emphasizes that women should be able to access midwifery-led care with midwives who have the competence to support the physiology of childbirth and one-to-one care.

Although Rooks’ paper (Rooks, 1999) was written some years ago, the two discourses are still relevant and ongoing in the concerns of the increasing rates of caesarean section and obstetric interventions in childbirth. In countries with midwifery-led care, main benefits have been proven, such as reductions in epidurals, episiotomies, and instrumental births, compared to models of medical-led care or shared care, without compromising safety (Shaw et al., 2016).

Moreover, the WHO (1996), has developed guidelines for care during childbirth where normal births are promoted and it is emphasized that women should give birth in places where they feel safe and are able to access appropriate care. Historically, home and hospital institutions have been the places of birth. Although births occur in different hospital settings, such as home-like birth centres, midwifery-led birthing units, and in high intervention hospital birthing facilities. Most of the studies on birthplace has focused on studying the effects of place on the perinatal and maternal outcomes, and the interventions in labour (Brocklehurst et al., 2011; Davis et al., 2011). Findings suggest that planning the place of birth has a significant influence on mode of birth, rates of intrapartum intervention, and on birth experiences (Brocklehurst et al., 2011; Davis et al., 2011; Lindgren, Brink, & Klinberg-Allvin, 2011; Murray-Davis et al., 2012). In a recently published review study undertaken to inform WHO intrapartum guidelines of what matter for women during childbirth, environment of care and the atmosphere of the local facility was highlighted (Downe, Finlayson, Oladapo, Bonet, & Gulmezoglu, 2018).

The environment has been considered a meta-concept in nursing since the time of Florence Nightingale (Andrews, 2003; Nightingale, 1859) and already in 1993, Kearns argued that people ascribed meaning to places and spaces where they received care. Kearns (1993) called for an increased acknowledgement of the association between place and health. This resulted in an interest in the concepts of place and space within the nursing and midwifery fields (Andrews, 2002; Andrews & Shaw, 2008; Liaschenko, 1994; Sharp, 1999). Place and space represent separate concepts that interact in a dynamic relationship and are very much interrelated. Place is considered to be both a physical, material site that is located geographically, as well as something that is experiential and socially constructed by a dynamic interplay between physical, individual, social and symbolic factors (Gieryn, 2000). That is, places have different meaning and value for different people due to experiences, memories, and associations, that are mutable over time (Gieryn, 2000). Space is conceptualized as a more abstract concept and can be understood as a physical and social landscape, which is imbued in everyday life (Soja, 1996). A space could be exemplified as perceived space that invisibly surround people´s bodies. Moreover, space is also conceived spaces, which refers to our knowledge of spaces, primarily produced by discourses of power and ideology constructed by professionals (Soja, 1996). Based on research underpinning place as important this paper draw on research from Health Geography. Thus, the aim of this critical interpretive synthesis was to analyse and synthesize the research where concepts of place and space in relation to childbirth have been studied.

## Method

This literature review used critical interpretive synthesis as a method to integrate qualitative studies into a conceptual understanding (Dixon-Woods et al., 2006). This enabled us to go beyond mere descriptions of the included papers and thus identify a conceptual construction (Grant & Booth, 2009). This CIS follows the iterative, reflexive approach, comprising the following phases: ^1.^ formulating the review question, ^2.^ searching for the literature, ^3.^ sampling, ^4.^ determining the quality, ^5.^ extracting data and conducting an interpretive synthesis (Dixon-Woods et al., 2006).

### Data collection

#### Formulating the review question

Our formulated review question was broad: “*what does the health geography concepts place and space mean when used in research papers that focus on birth?*” This broad question allowed the concept to emerge from the analysis of literature.

#### Searching for the literature

The search strategy included inclusion and exclusion criteria. Inclusion criteria were research papers published in peer review journals, reporting qualitative data. However, the concepts place and space are interrelated, therefore, papers pertaining both concepts place or space were sought in relation to childbirth. The papers should be written in the English language and available in electronic databases with no restrictions with regards to publishing year. Exclusion criteria were papers that focused solely on pregnancy or the period after birth, and papers using a quantitative methodology. Four electronic databases, CINAHL, Medline, PsycInfo and Sociological abstracts, were systematically searched during the period of 2018-07-05 to 2018-08-27, using MeSH terms, Thesaurus, and subject headings.

Search terms included “space” OR “place” OR “setting”. These were combined with different words related to childbirth, such as “labor”, “labour”, “birth*” and “parturition”. Moreover, since our aim was to find qualitative papers, search terms such as “qualitative” OR ‘interview*, were searched.

#### Sampling

**Figure 1.**
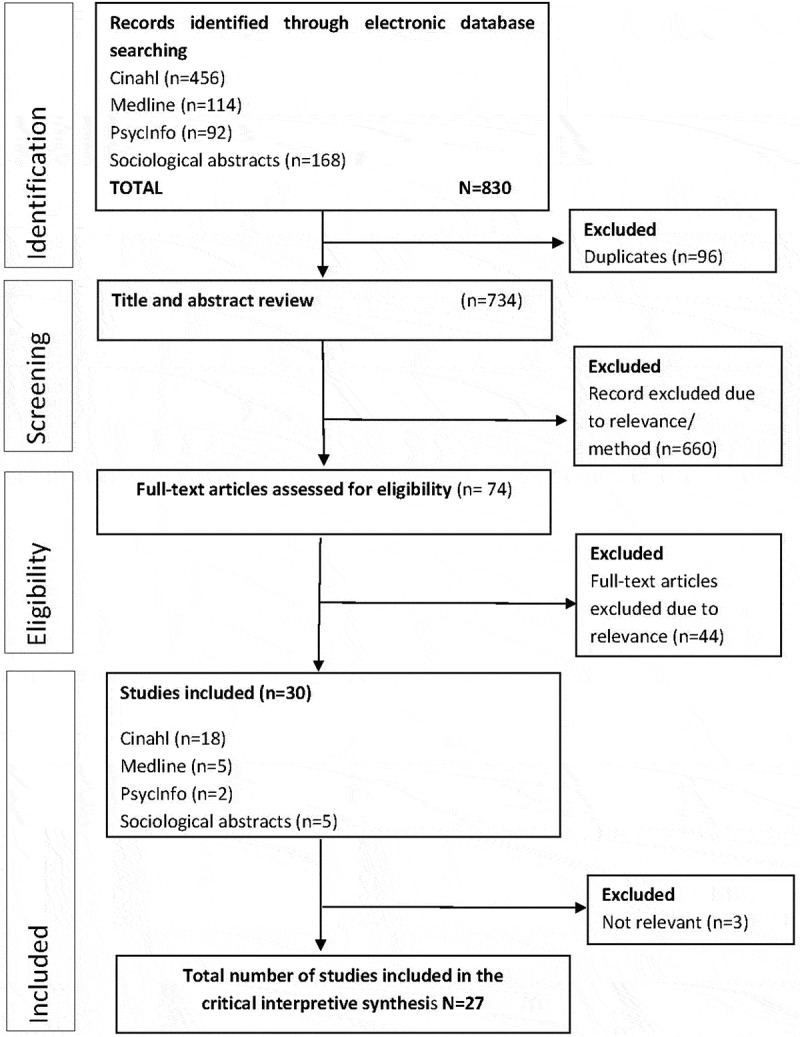
Flowchart of literature search and selection

One of the researchers conducted the database searches together with an experienced librarian (IMC, EF). The primary search strategy generated in total 830 papers identified by the electronic data base search and after removing duplicates, 734 papers remained and were selected and screened. Following assessments of abstracts, 74 full text papers were read and screened and this resulted finally in 27 papers, which were included in the analysis (Figure 1).

#### Determining the quality

The quality of each included paper was assessed by a quality rating template (SBU, 2017). The template is based on questions of study credibility (trustworthiness in the research findings), dependability (transparency in the method), confirmability (consistency between data and findings) and transferability (relevance of the research finding in other settings). The strengths of evidence in the template was graded according to quality; high, medium, or low quality. The quality was assessed independently by the first author (IMC) and the second author (IL), and the included papers had a level of medium or high quality.

#### Extracting data and conducting an interpretive synthesis

The initial analysis started by reading and summarizing the papers into matrices of each study. Each paper was read several times, and the findings sections were read line by line. Codes where identified, compared and pooled together. The analysis involved an iterative process of reading the papers and writing reflexive comments within the focus of CIS on understanding how a construct was conceptualized, studied and related to each other.

The first author identified the conceptual construction “creating a place for childbirth”, which conceptualized place and space in relation to childbirth. Thus, consistent with the approach described by Dixon-Woods et al. (2006), the analysis, and subsequent critical interpretation, were continuously developed based on reflexivity and dialogue between the authors. That is, exploration of the meaning of place and space in relation to childbirth.

## Findings

A total of 734 papers were screened, and 27 papers met the final inclusion criteria after assessment (Figure 1). The date range of publication for the results of the search was 1991–2018. Countries represented across the 27 papers were Australia (n = 11), UK (n = 5), Sweden (n = 3), New Zealand (n = 3), USA (n = 2), South Africa (n = 2), and Norway (n = 1) which are summarized in Table I. Data were collected through interviews (individual and focus groups) and observations (observations and filming of births). Two of the included papers used data from previous studies. One paper was a secondary analysis of previous interviews from two studies. Of the 27 papers, 13 included women’s voices as participants, 11 included midwives, and the remaining three papers included both women and midwives as participants (Table I).

**Table I.** Characteristics of studies included in the critical interpretive synthesis (N = 27)

Authors, year, country	Objective	Data collection	Design and data analysis	Findings
Abel and Kearns (1991), New Zealand.	To explore women´s opinions and experiences of home births.	Individual interviews with 6 women, who had experienced planned home births. Two primiparas, 4 multiparas.	Interpretive method with a feminist geography approach.	The place of birth women’s ability to assume control and to have continuity of care and continuity of place.
Bernhard et al (2014), USA.	To compare and contrast individual women´s home and hospital birth experiences.	Interviews in focus groups with 20 multipara who had had at least one hospital birth followed by at least one planned home birth.	A qualitative descriptive methodology with a qualitative content analysis.	Five themes emerged; Choices and empowerment, interventions and interruptions, disrespect and dismissal, birth space, connection.
Blix (2011), Norway.	to explore midwifery practice in homebirth settings in Norway especially practice assumed by the midwives to promote normal birth.	Individual or group interviews with 12 midwives who regularly attended home births.	Grounded theory.	The core category, avoiding disturbance meant that midwives prevented the woman from being disturbed and protected her from disturbance.
Borrelli et al. (2016), UK.	To conceptualize first time mothers expectations and experiences of a good midwife during childbirth in the context of different birthplaces.	Individual semi-structured interviews with 14 women before and after birth. The women had given birth in three different planned places of birth (home, Freestanding Midwifery Unit and Obstetric Unit).	Grounded theory.	A conceptual metaphor, the kaleidoscopic midwife illustrated how the midwife adapts to each woman´s individual needs in the context of each specific labour.
Borrelli et al. (2017), UK.	To explore first-time pregnant women´s expectations and factors influencing their choice of birthplace.	Individual semi-structured interviews with 14 women during pregnancy. The women had the option to give birth in three different birth settings. (home, Freestanding Midwifery Unit and Obstetric Unit).	Grounded theory.	Three main themes were identified; influencing factors on the choice of birthplace, expectations on the midwives being and doing roles, perceptions of safety.
Burns (2015), Australia.	To move the theoretical debate beyond the home/hospital dichotomy.	Individually interviews with 58 participants. 51were women who were pregnant and planning or had had a home birth in the last three years. 7 were professional doulas and independent midwives.	Burns (2015), Australia.	To move the theoretical debate beyond the home/hospital dichotomy.
Carlsson (2016), Sweden.	To generate a theory based on where women choses to be during the early labour process.	37 individual interview transcripts from 37 women who had given birth. 18 of these women had sought and been admitted to hospital during the latent phase of labour and 19 women had remained at home until active labour.	Carlsson (2016), Sweden.	To generate a theory based on where women choses to be during the early labour process.
Chadwick and Foster (2014), South Africa.	To study risk constructions in relation to choice of birth at home or via an elective Caesarean section.	Individual interviews with 24 pregnant women who were planning to undergo either a homebirth or an elective caesarean.	Discourse analysis.	Women choosing elective Caesareans positioned themselves within biomedical forms of knowing about childbirth. Choosing caesarean section was itself constructed as a form of risk management.
Coxon et al. (2014), UK.	To provide an understanding of what accounts for birthplace preferences.	Narrative interviews were performed with 41 pregnant women.	A prospective, longitudinal study with a narrative methodology.	Women who preferred to give birth at obstetric units (25 of 41 women) viewed birth as a medical risk. Those who planned birth in alternative settings also emphasized their intention and obligation to seek medical care if necessary.
Davis and Homer (2016), Australia.	To explore the way that birthplace impacts on midwives in Australia and the United King	12 midwives were interviewed by focus groups.	A qualitative descriptive study with a thematic analysis.	Place shapes midwives practice and the way they feel. Place also shapes the midwives ability to be with the woman.
Davis and Walker, (2010a), New Zealand.	To explore the way in which case-loading midwives in New Zealand construct midwifery and given these constructions to examine their practice within obstetric hospitals.	Individual interviews using semi-structured questions with 58 case-loading midwives.	A post structural feminist theoretical framework was used with NVIVO.	Midwives employ strategies to make space for childbirth. These include re-constructing the maternal body as a competent body, re-positioning the woman at centre of care, disrupting the obstetric gaze and creating an oasis of privacy, calm and women- centeredness within the birthing room.
Davis and Walker (2010b), New Zealand.	To explore the social production of space and place in relation to childbirth.	Individual interviews using semi-structured questions with 48 case-loading midwives.	Discourse analysis.	Places play a significant role in shaping midwives understanding of childbirth and thus their decision-making and clinical practice.
Hammond et al. (2014a), Australia.	To explore the impacts of physical and aesthetic design of hospital birth rooms on midwives.	6 filmed labours. 8 midwives participated in video-reflected interviews	A video ethnographic study with thematic analysis.	Midwives were strongly affected of the design of the birth rooms, which challenged the provision of care.
Hammond et al. (2014b), Australia.	To explore the relationship between the birth environment and the practice of midwifery using the theoretical approach of critical realism.	Individual interviews with 16 practising midwives.	An explorative descriptive methodology with thematic analysis.	Midwives cognitive and emotional responses indicated a relationship between hospital birth environments and midwifery practice.
Hammond et al. (2017), Australia.	To identify and describe the design characteristics of hospital birth rooms that support midwives and their practice.	21 face-to face photo-elicitation interviews with 16 midwives were conducted.	A qualitative descriptive methodology by the theoretical approach of critical realism with a thematic analysis.	Three design characteristics were identified that supported midwifery practice; friendliness, functionality and freedom.
Hastings-Tolsma et al. (2018), South Africa.	To describe the experience of women receiving care during childbirth.	Individually interviews with 12 women who recently had given birth.	A qualitative descriptive methodology with a thematic analysis.	Four themes were noted: Cocoon of compassionate care, personal regard for shared decision-making, beliefs about birth and protection
Kennedy et al. (2004), USA.	To expand knowledge of the process and outcomes of midwifery care	Individually interviews with 14 midwives and 4 recipients of midwifery care.	Narrative analysis.	Three themes were identified; the midwife in relationship with the woman, orchestration of an environment of care, and the outcomes of care.
Kuliukas et al. (2016), Australia.	To explore midwives experiences of intrapartum transfer.	Individual interviews with 17 midwives who cared for women in birth centres who were transferred to the tertiary obstetric unit during labour.	A descriptive phenomenological study.	Midwives find transfer in labour challenging both emotionally and practically. Midwives acknowledged the challenge of finding balance between fulfiling parents ‘birth plan wishes with hospital protocol and maintaining safety.
Lock and Gibb (2003), Australia.	To describe the power that place holds over the postnatal-care experiences of women.	Conversational interviews with 5 women birthing in hospital.	A phenomenology approach with thematic analysis.	Four major constructs of experience were revealed through analysis and include spatiality, corporeality, temporality and relationality.
Lee, Ayers, and Holden (2016), UK.	To examine decisions regarding place of birth among a group of high-risk pregnant women. The intention was to consider differences and similarities between the groups in the factors they considered and emphases they placed on these when deciding on place of birth.	26 participants with high-risk pregnancies, at least 32 weeks pregnant were interviewed with semi-structured questions. Half the participants were planning hospital births and half were planning homebirths.	Thematic analysis	Both groups were concerned about safety but they expressed different concerns. Women planning homebirths displayed faith in the natural birth process and stressed the quality of the birth experience. Women planning hospital births believed the access to medical care outweighed their misgivings about the physical environment.
Mondy et al. (2016), Australia.	To explore the concepts of domesticity within the birth space. The specific objectives were to explore, describe and compare birth spaces with different domestic characteristics and subsequently, how labouring women worked within these spaces during the labour process.	Filming of 6 births in two different birth locations (territory hospital n = 5 and a stand-alone birth centre n = 1). Video footage of women labouring at home was used to compare and contrast women’s experiences. 9 Interviews were performed with the women or their attended people and 8 interviews were performed with their midwives.	Video ethnography and reflexive interviewing analysed with latent content analysis.	In general women labouring in conventional hospital acted and interacted with the environment in a passive way. In essence, all but one of the women labouring and birthing in these spaces took on the role of a “patient”. Domestication of the space’ was the mechanism this woman used to retain a sense of ownership within the birth space.
Newburn (2012), UK.	To explore the model of care provided at a birth centre from the perspectives of midwives and parents.	114 hour of observation at the birth centre observing antenatal, intrapartum and postnatal care. 14 individual interviews with parents after birth (seven with women and seven with women and their partner together) and 11 interviews with staff (9 midwives and 2 maternity assistants).	An ethnographic study.	Most women and men at the Birth centre perceived it as offering the best of both worlds based on its proximity to the labour ward.
Nilsson (2014), Sweden.	To gain a deeper understanding of women’s negative experiences in the delivery room.	Individual interviews with 21 women (15 pregnant women with intense fear of childbirth, and six women who had experienced intense fear of childbirth 7–11 years prior to the interview).	A hermeneutic approach.	The delivery room was, for these women, a place creating fear of childbirth. The birth environments are understood as power structures, containing views of women’s birthing bodies as machines, and delivery rooms as surveillance environments.
Nilsson et al. (2010), Sweden.	To describe the meaning of previous experiences of childbirth in pregnant women who have exhibited intense fear of childbirth such that it has an impact on their daily lives.	Individual interviews with 9 pregnant women with intense fear of childbirth who were pregnant with their second child and considered their previous birth experiences negative.	A descriptive phenomenological study	The women felt as if they had no place in the delivery room, that they were unable to take their place and that even if the midwife was present, she did not provide support. The experience remained etched in the women’s minds and gave rise to feelings of fear, loneliness, and lack of faith in their ability to give birth and diminished trust in maternity care.
Parrat and Fahy (2004), Australia	To explore what affects birth space has on women´s birth experience and outcome and how can midwives provide a holistically safe birth place.	A total of 6 women who were considered “low risk” at the beginning of labour, participated in individual interviews. Three of the participants had homebirths experiencing continuity of care in partnership with a midwife. The remaining three participants had the fragmented care of medically managed childbirth in hospital.	Feminist constructivism with grounded theory.	A “holistically safe” space is jointly constructed by midwife and woman. This model enables the woman to feel in control of her birth space, respond intuitively and facilitate her potential for a safe, natural birth.
Seibold et al. (2010), Australia.	To explore and describe midwives perceptions of birth space and clinical risk management and their impact on practice both before and after a move to a new facility.	18 midwives, including graduate year midwives, caseload midwives and hospital midwives working normal shifts, employed within a hospital were observed and interviewed in focus groups.	An explorative descriptive study utilizing a modified participatory approach.	Midwives desire to create the ideal birth space was hampered by a prevailing biomedical discourse which emphasized risk. Midwives in all three groups saw themselves as the gatekeepers, “holding the space” or “providing a bridge” for women, often in the face of a hierarchical hospital structure with obstetricians governing practice.
Townsend et al. (2016), Australia.	To describe midwives’ perceptions of the birth bed.	Individual interviews with 14 midwives from one Queensland maternity.	A qualitative descriptive design. Thematic analysis.	A common feature of the modern birth space is the bed. The themes highlight differences in how the midwives conceptualized the use of a bed within a birth space. While some avoided the use of the bed altogether others would only conceive of women moving off the bed if everything was “normal”. How the bed was culturally constructed appeared to dictate clinical practice.

Our critical interpretive synthesis generated a conceptual construction comprising four synthetic constructs, which together explained the concepts of place and space in relation to childbirth.

### The conceptual construction- creating a space for childbirth

The most prominent and comprehensive conclusion in this literature study’s analysis was the need for creating a space for childbirth—a birthing space that was more than a welcoming physical space. This space positioned the woman at the centre of the childbearing experience, supported her needs, desires, and the philosophy of birthing that the woman brought with her (Bernhard, Zielinski, Ackerson, & English, 2014; Borrelli, Spiby, & Walsh, 2016; Davis & Walker, 2010a; Hammond, Homer, & Foureur, 2014a; Kennedy, Shannon, Chuahorm, & Kravetz, 2004; Seibold, Licqurish, Rolls, & Hopkins, 2010). According to woman´s philosophy of birthing, the midwife established an atmosphere that also supported the art and philosophy of the midwives (Blix, 2011; Borrelli et al., 2016; Kennedy et al., 2004). This meant that the midwives were holding the space with professional knowledge and keeping the process safe with normalcy preserved (Abel & Kearns, 1991; Borrelli et al., 2016; Carlsson, 2016; Chadwick & Foster, 2014; Davis & Homer, 2016; Davis & Walker, 2010a; Hastings-Tolsma, Nolte, & Temane, 2018; Kennedy et al., 2004; Lock & Gibb, 2003; Seibold et al., 2010). However, there was a need for the midwives to have an awareness of the power of the place. A power that was due to hinder cultural norms, policies, and different models of care, and exercised through social interrelations by health care professionals, managers in the health care system, and other people involved in childbirth (Davis & Walker, 2010a, 2010b; Kennedy et al., 2004; Kuliukas, Lewis, Hauck, & Duggan, 2016). The created space was protected by a boundary to the birthing room (Burns, 2015; Chadwick & Foster, 2014). The door to the room was kept closed and guarded by the midwife from intrusion (Davis & Walker, 2010a). Keeping the door closed symbolized a physical boundary, hindering other professions or persons from barging in and intervening in the birth process (Blix, 2011; Burns, 2015; Chadwick & Foster, 2014; Davis & Walker, 2010a; Parratt & Fahy, 2004; Seibold et al., 2010). The door also protected the woman and the midwife from external noise (Blix, 2011; Davis & Homer, 2016) or stress caused by activities from the workload at the department (Davis & Walker, 2010a; Hammond, Homer, & Foureur, 2017). Within the door, the midwives situated themselves with the woman, creating a space for childbirth (Borrelli et al., 2016; Davis & Walker, 2010a; Seibold et al., 2010). The created space consisted of four different prominent spaces; homely, spiritual, safe, and territorial spaces, which all affected childbirth.

### A homely space

A homely space was characterized by a place where the woman didn´t have to adapt to the environment (Abel & Kearns, 1991; Hammond et al., 2014a; Lock & Gibb, 2003; Mondy, Fenwick, Leap, & Foureur, 2016). This meant no problem when the birth took place in the women’s own homes where a sense of familiarity, freedom and self-confidence occurred (Abel & Kearns, 1991; Bernhard et al., 2014; Borrelli, Walsh, & Spiby, 2017; Carlsson, 2016; Coxon, Sandall, & Fulop, 2014; Lee, Ayers, & Holden, 2016; Lock & Gibb, 2003; Parratt & Fahy, 2004). By contrast, entering hospital brought the women into a strange place with design characteristics of an emergency hospital room, uncomfortable and signified by the nature of the bed placed in a central position (Davis & Homer, 2016; Davis & Walker, 2010b; Hammond et al., 2014a; Lock & Gibb, 2003; Mondy et al., 2016; Newburn, 2012; Townsend, Fenwick, Thomson, & Foureur, 2016). This strange place forced the women to adapt and thus, most women interacted with the environment in a passive way (Davis & Walker, 2010a, 2010b; Mondy et al., 2016; Townsend et al., 2016). The environments design and the equipment at the hospital signalled what would happen in the room, which was danger and abnormality. This affected both the woman and the midwife (Davis & Walker, 2010b; Hammond et al., 2014a, 2017). The midwife was the one who had the opportunity and authority to change the birthing room to a homelier place. This was done by modifying the lightning and re-arranging the room, putting the bed at the side and thus providing space and encouraging the woman to move around (Davis & Homer, 2016; Davis & Walker, 2010a, 2010b; Hammond et al., 2014a; Parratt & Fahy, 2004; Townsend et al., 2016). Furthermore, the midwives encouraged the women to surround themselves with their own familiar things, making them feel free to adjust the labour space according to personal needs, bringing their homes to the hospital (Davis & Walker, 2010a; Hammond et al., 2014a; Mondy et al., 2016; Newburn, 2012; Parratt & Fahy, 2004). However, sometimes this “nest” had consequences for the midwife who no longer had a place for performing her job (Hammond, Foureur, & Homer, 2014b). Lack of space meant that the midwives were less likely to remain in the birthing room (Hammond et al., 2014b). In essence, a homely space contributed to a feeling of being at home, a non-threatening, comfortable relaxing space for the women, which implied a sense of belonging (Lock & Gibb, 2003; Newburn, 2012).

Moreover, a homely space facilitated the women´s confidence, self-agency, and to take an active role in their care, thus taking more control and enabling them to be a conductor of their own birth experience (Abel & Kearns, 1991; Coxon et al., 2014; Lee et al., 2016; Lock & Gibb, 2003; Mondy et al., 2016; Newburn, 2012). Finally, the design of hospital birth rooms also affected the midwives (Hammond et al., 2014b; Townsend et al., 2016). A sense of homeliness meant a sense of normality, which was in line with midwifery promoting normal birth. When the midwife had to leave her area of familiarity, the hospital or the home, and go to an area of which she was less acquainted, this could be challenging and raise feelings of being out of the comfort zone and out of place (Kuliukas et al., 2016).

### A spiritual space

A spiritual space was a place where the woman could withdraw, that was peaceful, calm and silent, a nice place to be in (Bernhard et al., 2014; Blix, 2011; Davis & Homer, 2016; Davis & Walker, 2010a; Hammond et al., 2014a; Parratt & Fahy, 2004). Being able to withdraw and enter an inner world and remain in one´s own space enabled the woman to be present in herself, and thus, present in the room, “being fully there” (Bernhard et al., 2014; Blix, 2011; Chadwick & Foster, 2014; Nilsson, Bondas, & Lundgren, 2010). This helped the woman to connect to her own body (Bernhard et al., 2014; Chadwick & Foster, 2014) and able to concentrate on and follow the process of birth (Blix, 2011; Davis & Homer, 2016; Davis & Walker, 2010a). Being present created feelings of actively participating in the process and that the birth was in progress. A spiritual space was also conceptualized as a space produced by human activity (Hammond et al., 2017), a space with others, and a space of trust, with a cocoon of compassionate and support (Abel & Kearns, 1991; Bernhard et al., 2014; Hastings-Tolsma et al., 2018; Parratt & Fahy, 2004).

Continuity was regarded as important and continuity of place meant that no transfers was performed and that the birth could progress without interruptions (Abel & Kearns, 1991; Bernhard et al., 2014). Moreover, continuity of care facilitated trust and involved having a relationship with a supportive midwife that was available, and had faith in the woman’s ability to give birth (Abel & Kearns, 1991; Bernhard et al., 2014; Borrelli et al., 2017; Kuliukas et al., 2016; Parratt & Fahy, 2004; Seibold et al., 2010). Continuity of care was of outmost importance when the woman had to transfer to another birthplace (Kuliukas et al., 2016).

### A safe space

A safe space was a major consideration for the women regardless of where birth took place (Burns, 2015; Lee et al., 2016; Parratt & Fahy, 2004). Safety was conceptualized as both physical and emotional safety. Physical safety was described as knowing that the midwives and doctors who attended them held expertise and possessed theoretical knowledge, and professional competences. Physical closeness was important—being there, available if needed (Blix, 2011; Borrelli et al., 2016, 2017; Carlsson, 2016; Coxon et al., 2014; Davis & Homer, 2016; Lock & Gibb, 2003; Parratt & Fahy, 2004).

The hospital itself was acknowledged as a place of safety, reassurance, and a controlled environment (Borrelli et al., 2016, 2017; Carlsson, 2016; Coxon et al., 2014; Davis & Homer, 2016; Lock & Gibb, 2003; Townsend et al., 2016). A controlled environment included midwives as machine watchers, monitoring the wellbeing of mother and baby, assessing the progress of labour, and providing the necessary care and support to facilitate a safe and satisfying labour and birth. At the same time, it means observing without disturbance (Blix, 2011; Davis & Homer, 2016; Townsend et al., 2016) and having the knowledge to understand when to intervene and, if needed, having a rapid access to medical care (Borrelli et al., 2017; Carlsson, 2016; Coxon et al., 2014; Davis & Homer, 2016; Lee et al., 2016; Lock & Gibb, 2003; Newburn, 2012; Seibold et al., 2010; Townsend et al., 2016). A safe space also included emotional safety (Lee et al., 2016), i.e. having someone providing a safe space for the woman, and just being present with her (Borrelli et al., 2016; Hastings-Tolsma et al., 2018; Parratt & Fahy, 2004; Seibold et al., 2010), knowing that those who were in the birthing room had a presence and cared for the woman´s wellbeing (Abel & Kearns, 1991; Bernhard et al., 2014; Blix, 2011; Hastings-Tolsma et al., 2018; Lee et al., 2016; Newburn, 2012; Parratt & Fahy, 2004). It also included people that the women had chosen to surround themselves with (Bernhard et al., 2014; Carlsson, 2016; Hastings-Tolsma et al., 2018). A “holistically safe” space was jointly constructed by the midwife and woman, which enabled the woman to feel safe, meaning they could release their mental control (Parratt & Fahy, 2004).

### A territorial space

The birthing place could be described as a territory, sometimes with a hierarchical power structure and an authority of the institution where the birth took place (Davis & Homer, 2016; Lock & Gibb, 2003; Nilsson, 2014; Nilsson et al., 2010; Seibold et al., 2010). Ideally, the woman should govern the space during childbirth. If this ideal state appeared, then the woman had the ownership of the space (Townsend et al., 2016). This meant that she didn´t become a patient or needed to take the role of a patient, which is a powerless position (Abel & Kearns, 1991; Bernhard et al., 2014; Lee et al., 2016; Lock & Gibb, 2003; Mondy et al., 2016; Newburn, 2012; Nilsson et al., 2010; Seibold et al., 2010; Townsend et al., 2016). Owning the space was often enabled when the birth took place in the women´s own homes, which was an empowering place (Bernhard et al., 2014). In contrast, when birthing at a hospital, the space was everyone’s space, and the space was described as only “lent” to the women (Seibold et al., 2010). This meant that the hospital maintained control over the space, with unspoken roles of the institution, which could imply a higher risk of medical interventions (Abel & Kearns, 1991; Bernhard et al., 2014; Burns, 2015; Chadwick & Foster, 2014; Davis & Homer, 2016; Seibold et al., 2010). An important function that contributed to the woman retaining a sense of ownership of the birth space was positioning her at the centre of the care (Davis & Walker, 2010a; Hastings-Tolsma et al., 2018; Newburn, 2012; Parratt & Fahy, 2004). By acknowledging that the woman brought a knowledge base with her, a shared mutual understanding was achieved where the woman was confirmed as a person owning the space (Hastings-Tolsma et al., 2018; Kennedy et al., 2004).

This recognition in line with respecting and responding to the woman´s preferences was essential (Burns, 2015; Davis & Walker, 2010a; Hastings-Tolsma et al., 2018; Kennedy et al., 2004). As well as “meeting the woman where she was”, it individualized the care, supporting and guiding her on her own terms (Hastings-Tolsma et al., 2018; Kennedy et al., 2004).

Moreover, strengthening the woman to take an active part in shared decision-making emerged as foundational for the midwife’s relationship with the woman during childbirth

(Chadwick & Foster, 2014; Davis & Walker, 2010a; Hastings-Tolsma et al., 2018; Kennedy et al., 2004; Parratt & Fahy, 2004). The opposite was experienced when the woman was ignored with feelings of being dehumanized and faceless. Examples include when the providers focused more on the uterus than on her as a whole person, a lack of information, or, even worse, if the woman was disrespected (Bernhard et al., 2014; Nilsson, 2014; Nilsson et al., 2010). Furthermore, childbirth could be a threat to bodily integrity with loss of bodily control by leaking bodies, tearing of the body, and losing control by making noises. Bodily boundaries preserved dignity regardless of place of birth (Burns, 2015; Chadwick & Foster, 2014) and respected boundaries protected privacy and intimacy (Burns, 2015; Davis & Homer, 2016; Newburn, 2012).

### Discussion

This study explored the concepts of place and space related to childbirth and brings together research on 27 papers in a critical interpretive synthesis. To our knowledge, this is the first review to explore the health geographic concepts in relation to childbirth. Geographical explorations highlighted that a birthing space had to be created in a mutual relationship between the woman and the midwife. This space should be women-centred (Bernhard et al., 2014; Borrelli et al., 2016; Kennedy et al., 2004) and protected by a boundary, hindering intrusion from others to preserve normality (Blix, 2011; Burns, 2015; Chadwick & Foster, 2014; Davis & Walker, 2010a; Parratt & Fahy, 2004; Seibold et al., 2010). Our findings are in line with the midwifery model developed by Berg, Olafsdottir, and Lundgren (2012). This midwifery model emphasizes the importance of creation of a birthing atmosphere that strengthening and supports normalcy (Berg et al., 2012). It may be concluded that the space in which midwifery practice care occurs shape the nature of that practice and preserve normality by focusing on normality (Berg et al., 2012; Dahlberg et al., 2016). However, different power constructions are in the place of birth, especially when the birth takes place within a hospital (Berg et al., 2012; Davis & Homer, 2016; Lock & Gibb, 2003; Nilsson, 2014; Nilsson et al., 2010; Seibold et al., 2010). The power is exercised through hierarchical structures and social interrelations (Berg et al., 2012; Davis & Walker, 2010a; Kennedy et al., 2004; Kuliukas et al., 2016).

This study demonstrated that the midwives tried to keep the door closed to the birthing room, hindering other professions or persons from barging in and intervene in the birth process (Blix, 2011; Burns, 2015; Chadwick & Foster, 2014; Davis & Walker, 2010a; Parratt & Fahy, 2004; Seibold et al., 2010). When midwives independently facilitate women-centred care and remain continuously present in the birthing room, this reduces not only the number of people involved, but most importantly, this also promotes normal birth (Berg et al., 2012; Bohren, Hofmeyr, Sakala, Fukuzawa, & Cuthbert, 2017). Women allocated to continuous support are more likely to have a spontaneous vaginal birth and less likely to have a caesarean birth or instrumental vaginal birth (Bohren et al., 2017). Thus, working behind a closed door prevents midwifery from becoming visible to other professions, who might feel excluded, and this affects how cooperation takes place at the clinical ward (Hansson, Lundgren, Hensing, & Carlsson, 2019). It may be assumed that the women’s birth experiences are affected by such dissonance in the team. Hansson et al (2019) states that these power structures need to be problematized on an organizational level to promote teamwork around the childbearing woman. Against a backdrop of the milieu of birthing in the developed world, different discourses compete for the safest birth (Walsh, 2010). Women´s planned place of birth is influenced by several complex factors, such as cultural and normative expectations and earlier experiences (Coxon, Sandall, & Fulop, 2015). Our study demonstrates that a safe place was a major consideration, regardless of which location birth took place (Burns, 2015; Lee et al., 2016; Parratt & Fahy, 2004). This finding is in line with the recently published review of Downe et al. (Downe et al., 2018) that highlighted that women have a strong desire for safe care during childbirth. It´s of utter importance to be aware that the woman´s understanding of birth risk and safety do not always align well with clinical risk assessments (Coxon et al., 2015).

The findings in this study describes four important spaces: homely, spiritual, safe, and territorial spaces, in relation to childbirth. These findings are confirmed by Fahy and Parrats theory describing the birth terrain (Fahy & Parratt, 2006). In their theory, concepts such as sanctum is used to define a homely space, and the sub-concept surveillance room could be linked to our two spaces, safety space and spiritual space (Fahy & Parratt, 2006). Finally, Fahy and Parrat (Coxon et al., 2015) used the concept jurisdiction, meaning, “having power to do as one wants”. This is similar to our finding of the territorial space emphasized by the woman’s need to own the space during childbirth (Abel & Kearns, 1991; Bernhard et al., 2014; Lee et al., 2016; Lock & Gibb, 2003; Mondy et al., 2016; Newburn, 2012; Nilsson et al., 2010; Seibold et al., 2010).

It is concluded that childbirth is an issue that encompasses more than the environment, the meta-concept in nursing, and that studies in reproductive health will benefit greatly from using geographical perspectives. Taken together, place and space are concepts that have pivotal connections to childbirth (England, Fannin, & Hazen, 2019).

## Methodological considerations

The trustworthiness in reporting syntheses of qualitative research must be rigorous (Tong, Flemming, McInnes, Oliver, & Craig, 2012). This critical interpretive synthesis attempts to be transparent by following the analysis according to Dixon-Woods et al. (2006). The systematic literature search of four databases conducted together with an experienced librarian and careful selection of relevant papers strengthen the credibility of the study. Dependability was also strengthened by the systematic and transparent data collection in several steps, including independent quality assessments of the included papers by the authors. In addition, only papers with medium or high quality were included in this study. In the data analysis process, there was a dialogue between the authors in order to not miss anything essential in the included papers. The large numbers of included papers with comprehensive content that corresponded to the purpose of this study also contributes to the confirmability of the findings. The transferability has to be seen in the light of the included papers, covering seven countries in different parts of the world, although only from middle- and high-income countries.

## Conclusion

This critical interpretive synthesis demonstrates a conceptual construct of a need to create a space for childbirth that is underpinned by four essential aspects of space; a homely space, a spiritual space, a safe space, and a territorial space. Within this perspective of conceptual understanding of the importance of place and space in relation to childbirth, it is suggested that the locations where childbirth takes place are imbued with cultural and personal meanings, and are products of discourse, which influences how care is perceived, given and received. The midwifery care will, therefore, provide more optimal prerequisites for the childbirth if space is created in order to consider the preferences of the women. The birth should be able to progress in a calm and safe place without interruptions with space controlled by the women with continuity in relation to the midwife. Thus, the findings of this study suggest midwives to strive to establish an atmosphere of self-determination, confidence, and familiarity for the women in order to increase a shared-decision making and autonomy. We propose that the findings from this review will provide a useful dialogue in midwifery education and in clinical settings.
